# The effectiveness of post-processing head and neck CT angiography using contrast enhancement boost technique

**DOI:** 10.1371/journal.pone.0284793

**Published:** 2023-04-20

**Authors:** Chuluunbaatar Otgonbaatar, Pil-Hyun Jeon, Jae-Kyun Ryu, Hackjoon Shim, Sang-Hyun Jeon, Sung Min Ko, Hyunjung Kim, Jin Woo Kim

**Affiliations:** 1 Department of Radiology, College of Medicine, Seoul National University, Seoul, Republic of Korea; 2 Department of Radiology, Wonju Severance Christian Hospital, Yonsei University Wonju College of Medicine, Wonju, Republic of Korea; 3 Medical Imaging AI Research Center, Canon Medical Systems Korea, Seoul, Republic of Korea; 4 ConnectAI Research Center, Yonsei University College of Medicine, Seoul, Republic of Korea; Universiti Sains Malaysia, MALAYSIA

## Abstract

**Background and purpose:**

This study aimed to investigate the potential of contrast enhancement (CE)-boost technique in the head and neck computed tomography (CT) angiography in terms of the objective and subjective image quality.

**Materials and methods:**

Consecutive patients who underwent head and neck CT angiography between May 2022 and July 2022 were included. The CE-boost images were generated by combining the subtracted iodinated image and contrast-enhanced image. The objective image analysis was compared for each image with and without CE-boost technique using the CT attenuation, image noise, signal-to-noise-ratio (SNR), contrast-to-noise-ratio (CNR), and image sharpness (full width at half width maximum, FWHM). The subjective image analysis was evaluated by two independent experienced radiologists in the following aspects: the overall image quality, motion artifact, vascular delineation, and vessel sharpness.

**Results:**

A total of 65 patients (mean age, 59.48 ± 13.71 years; range, 24–87 years; 36 women) were included. The CT attenuation of the vertebrobasilar arteries was significantly (*p* < 0.001) higher in the images obtained using CE-boost technique than in conventional images. Image noise was significantly (*p* < 0.001) lower for CE-boost images (6.09 ± 1.93) than for conventional images (7.79 ± 1.73). Moreover, CE-boost technique yielded higher SNR (64.43 ± 17.17 vs. 121.37 ± 38.77, *p* < 0.001) and CNR (56.90 ± 18.79 vs. 116.65 ± 57.44, *p* < 0.001) than conventional images. CE-boost resulted in shorter FWHM than conventional images (*p <* 0.001). Higher subjective image quality scores were also demonstrated by the CE-boost than images without CE-boost technique.

**Conclusions:**

In both objective and subjective image analysis, the CE-boost technique provided higher image quality without increasing the flow rate and concentration of contrast media in the head and neck CT angiography. Furthermore, the vessel completeness and delineation were superior in CE-boost images than in conventional images.

## Introduction

Head and neck computed tomography (CT) angiography is a widely used imaging tool for the identification of craniocervical vascular diseases [1,2]. In particular, cerebral CT angiography plays an important role in ischemic stroke, cerebral aneurysm, and cerebral arteriovenous malformation diagnosis due to its rapid examination and high accessibility [3,4]. However, the visualization of small intracranial vessels is sometimes not well demonstrated by the CT angiography and requires invasive digital subtraction angiography (DSA) for anatomical variation identification [5,6]. To clearly visualize small intracranial vessels, various methods (deep learning reconstruction, ultra-high-resolution CT, etc.) have been introduced to accurately visualize small cerebral vessels on CT angiography [7,8]. Deep learning image reconstruction increases both quantitative and qualitative image analysis on CT angiography compared with other image reconstructions by training images through deep convolutional neural networks [9,10]. Ultra-high-resolution CT has advantages to provide better image quality due to its higher spatial resolution with its smaller detector size compared with conventional CT [10].

The direct relationship between flow rate, concentration of iodinated contrast media, and higher vascular enhancement is well investigated [11,12]. Reduction of the rate and concentration of iodinated contrast media is crucial for patient safety [13]. For patients with chronic kidney disease, the amount of the iodinated contrast media should be as low as reasonably achievable [14]. Therefore, obtaining images with sufficient vascular enhancement using the lower flow rate and concentration of iodinated contrast media is required in the CT angiography without decreasing the image quality.

Without increasing the flow rate or concentration of the iodinated contrast media, the contrast enhancement (CE)-boost is a new post-processing technique for boosting vascular enhancement. This technique also provides higher image quality than conventional imaging technique [15]. However, the impact of the CE-boost technique in the head and neck CT angiography has yet to be assessed. In this study, we hypothesized that application of the CE-boost technique could improve both quantitative and qualitative image analysis by increasing CT attenuation and decreasing image noise compared with the conventional image. Therefore, the possibility of CE-boost technique in the head and neck CT angiography was evaluated in terms of objective and subjective image quality.

## Materials and methods

### Patient population

This retrospective study was approved by the Institutional Review Board, and all patients provided informed consent. From May 2022 to July 2022, 65 patients (mean age, 59.48 ± 13.71 years; range, 24–87 years; 36 women) who underwent head and neck CT angiography in our institution for various reasons including routine check-up, follow-ups for the aneurysm and/or intravascular diseases were reviewed. Patients with a prior allergic reaction to iodinated contrast material, who are pregnant, and who have an impaired renal function (glomerulation filtration rate < 30 mL/min/1.73m^2^) were excluded. The body mass index was 24.13 ± 2.85 kg/m^2^. Of these patients, there are 11 patients (16.92%) with diabetic mellitus, 20 (30.77%) with arterial hypertension, 13 (20%) with history of smoking, and 16 (24.61%) with hypercholesteremia.

### Scan protocol

All CT was obtained using a 320-MDCT volume scanner (Aquilion ONE PRISM, Canon Medical Systems Corporation). Tube voltage was 100 kVp and automatic tube current modulation was 80–550 mA. The gantry rotation time was 0.28 s; the detector collimation was 130 x 0.5 mm; the field of view was 320 mm; the matrix was 512 x 512; slice thickness was 0.5 mm; and the pitch was 0.813 and with helical scanning. Automatic bolus-tracking program (^SURE^Start, Canon Medical Systems Corporation) in the aortic arch (trigger threshold was 250 Hounsfield unit [HU]) was used for the scanning. A single bolus of 80 mL iodinated contrast medium (Iobitridol 350 mg, Guerbet, Aulnay-sous-Bois, France) was injected through the antecubital vein with a flow rate of 4.5 mL/s followed by a 30 mL saline with the same flow rate via a dual-head power injector (Stellant, MedRAD Inc., Warrendale, USA). The image was reconstructed with deep learning reconstruction with body sharp option (AiCE, Advanced Intelligent Clear IQ Engine, Canon Medical Systems Corporation). The CT angiography images were sent to a workstation for analysis (Vitrea, Vital, Minneapolis, USA).

### Contrast enhancement (CE)-boost images

**Fig 1:**
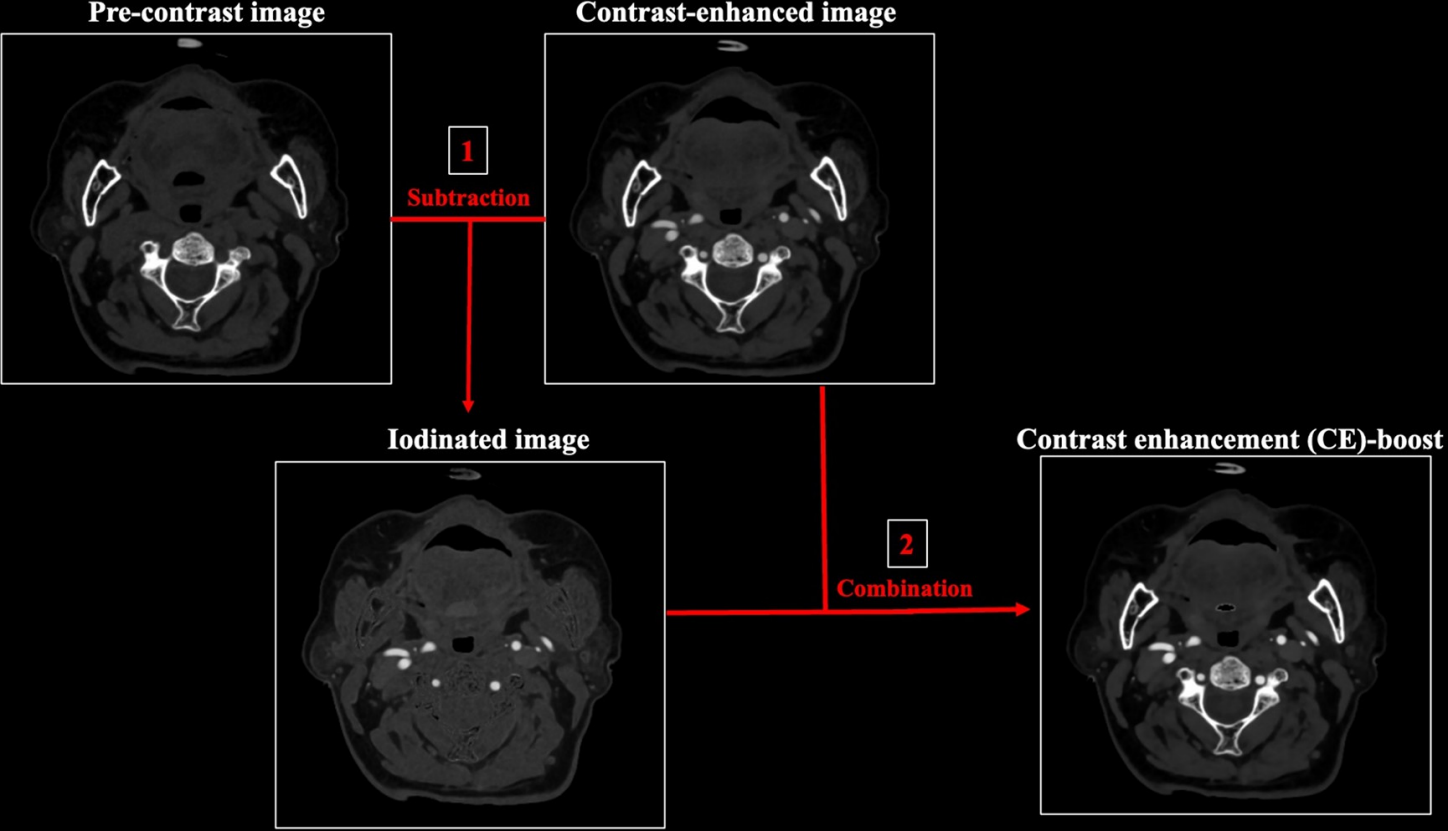
The flowchart of the CE-boost technique. The iodinated image was obtained by subtracting the contrast-enhanced image from the pre-contrast image (1). The contrast enhancement (CE)-boost images were then generated by combining the subtracted iodinated image and contrast-enhanced image through automatic denoising procedure (2).

The iodinated image was obtained by subtracting the contrast-enhanced image from the pre-contrast image. The contrast enhancement (CE)-boost images were then generated by combining the subtracted iodinated image and contrast-enhanced image through automatic denoising procedure (Fig 1). This technique has been developed to increase the contrast in CT images without requiring additional hardware.

### Objective image analysis

In each patient, an objective analysis was evaluated by a radiologist with 4 years of experience. The CT value of the precommunicating (P1)/postcommunicating (P2) segments of the posterior cerebral artery (PCA), basilar artery (BA), and four segments (pre-foraminal, foraminal, extradural, and intradural segments) of the vertebral arteries (VA) was measured. The image noise was determined by the standard deviation (SD) of the CT value by placing a region of interest (ROI, 100 mm^2^) in the subcutaneous fat. The SNR was measured by dividing each CT value of the arteries by the SDs. The CNR was calculated as the difference between the CT value of the vessels and the trapezius muscle, which was then divided by the image noise. The largest possible size of the ROIs at the center of the vessels was performed depending on the diameter of each vessel while avoiding the inclusion of the surrounding bone, calcification, and vessel wall. The image sharpness was evaluated using full width at half width maximum (FWHM) with ImageJ software (The National Institutes of Health, Maryland) and OriginPro 2022b (OriginLab Corp., Northampton, MA) [16]. The CT attenuation profile was generated with a horizontal line in the central axis of P1 segment of PCA (FWHM_pca_), BA (FWHM_ba_), and VA (FWHM_va_). The FWHM of each vessel were obtained from the CT attenuation profile. The shorter FWHM indicates a higher sharpness.

### Subjective image analysis

Two independent experienced radiologists (with 3 years and over 20 years of experience in diagnostic radiology) evaluated the subjective image analysis. The radiologists were blinded to the images and randomly evaluated the image quality by using a five-point Likert scale in the following aspects: overall image quality, motion artifact, vascular delineation, and vessel sharpness [17,18] (Table 1).

**Table 1:** Subjective image analysis scoring.

Score	Image quality	Motion artifact	Vascular delineation	Vessel sharpness
5	Excellent	No artifact	Excellent vessel definition	Excellent
4	Good	Few artifacts	Good vessel definition	Good
3	Satisfactory	Some artifacts	Moderate vessel definition	Moderate
2	Weak	Obvious	Poor vessel definition but sufficient for the diagnosis	Poor but acceptable
1	Poor	Severe	Poor vessel definition and not sufficient for the diagnosis	Poor and not acceptable

### Statistical analysis

Continuous variables were presented as the mean ± SD. Data normality was performed using the Kolmogorov–Smirnov test and Shapiro–Wilk tests. The CT attenuation, image noise, SNR, and CNR was compared for each image with and without CE-boost technique by using a paired-samples t-test, and FMHW, subjective analysis was compared with Wilcoxon signed-rank test. An inter-observer agreement analysis was performed using Cohen’s kappa (k) coefficient, whereby a k value of more than 0.81 was excellent, 0.61–0.8 was substantial, 0.41–0.6 was moderate, 0.21–0.40 was fair, and less than 0.21 was fair [19]. Statistical significance was set to p < 0.05. Data were analyzed using the SPSS statistical software ver. 25.0 (IBM, Armonk NY, USA).

## Results

**Fig 2:**
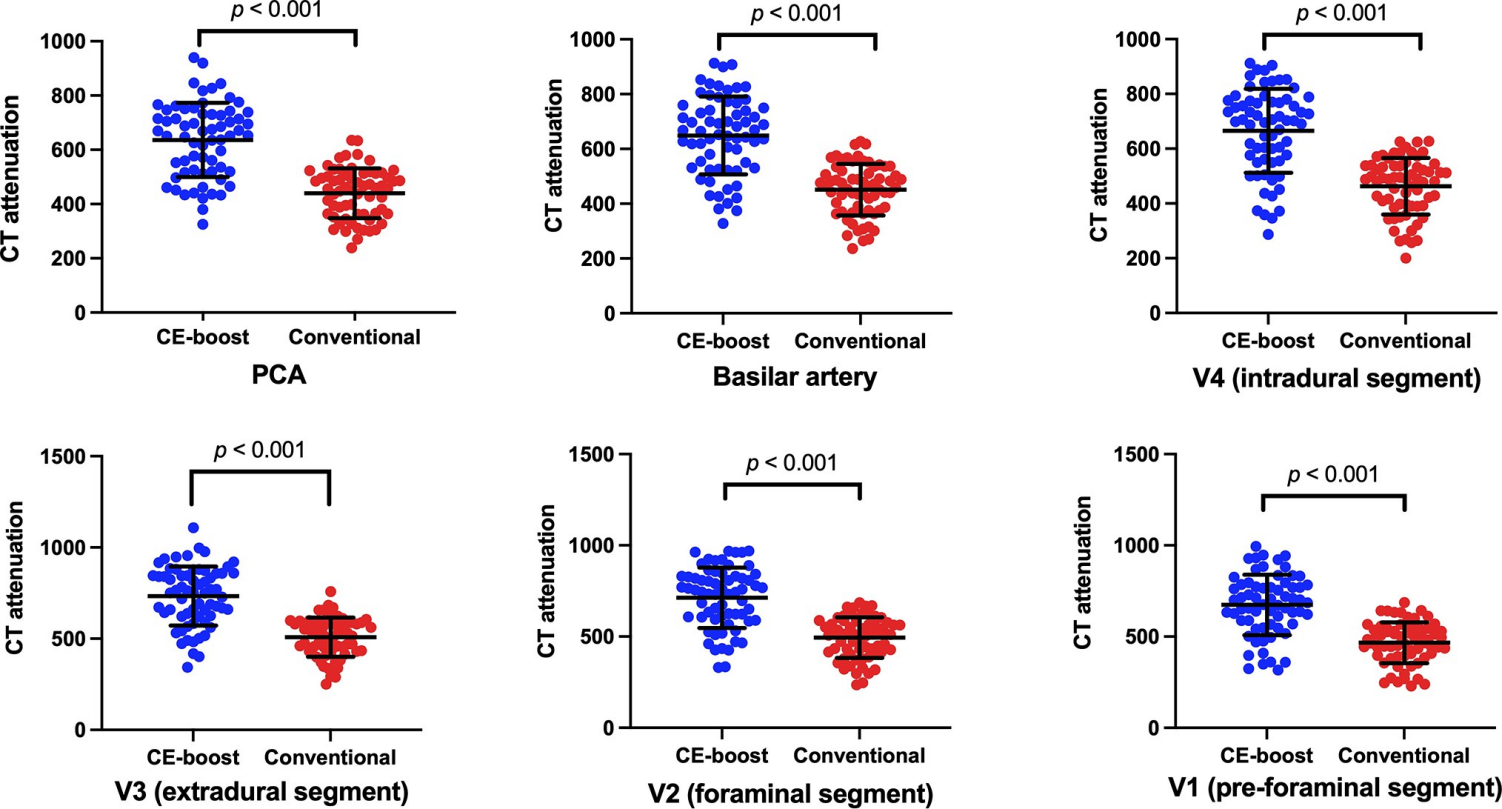
The CT attenuations between CE-boost and conventional images from the different anatomical regions. CE-boost technique resulted in significantly higher CT attenuation than the conventional images from the different anatomical regions including PCA, basilar artery, pre-foraminal segments of the vertebral artery, foraminal segments of the vertebral artery, extradural segments of the vertebral artery, and intradural segments of the vertebral artery.

**Fig 3:**
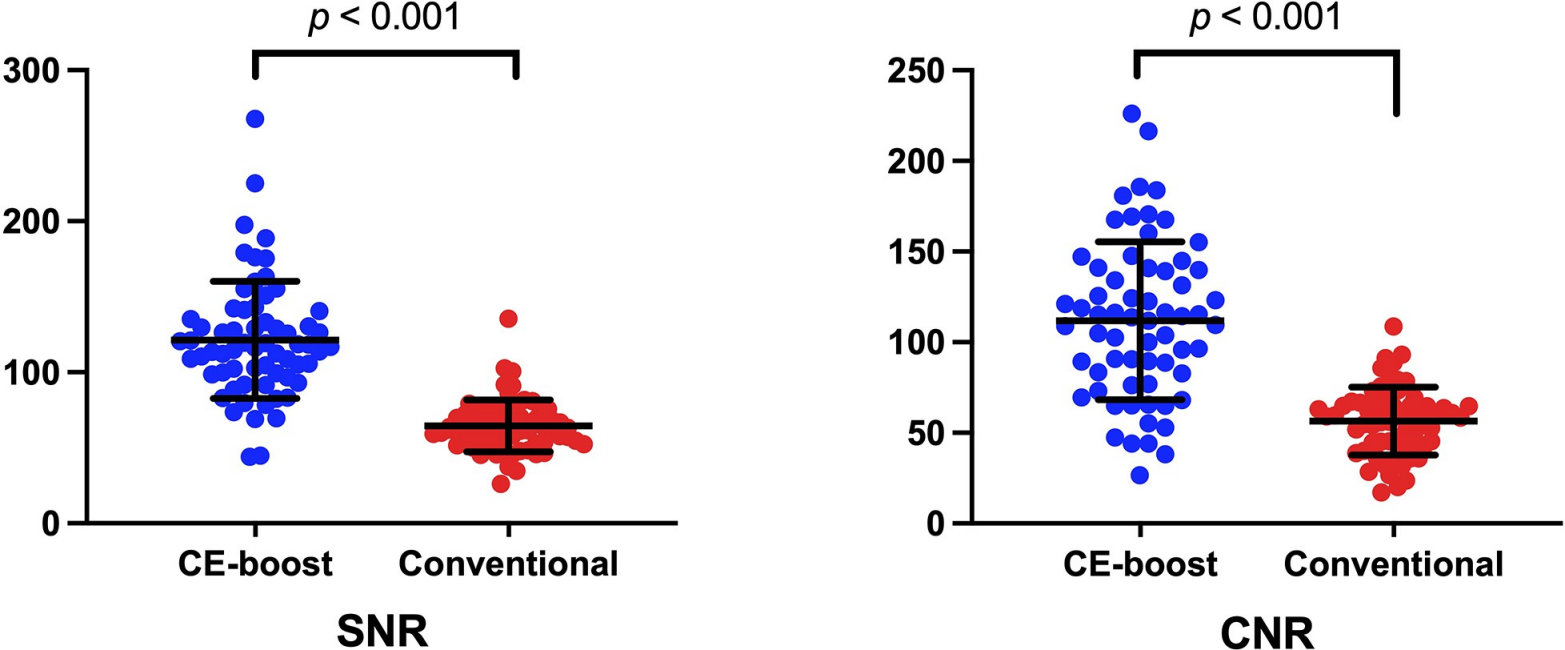
The results of SNR and CNR from the different anatomical regions. The mean value of SNR was significantly improved from 64.43 ± 17.17 with conventional images to 121.37 ± 38.77 with CE-boost, *p* < 0.001, while the mean value of CNR was also significantly increased 56.90 ± 18.79 with conventional image to 116.65 ± 57.44 with CE-boost, *p* < 0.001.

**Fig 4:**
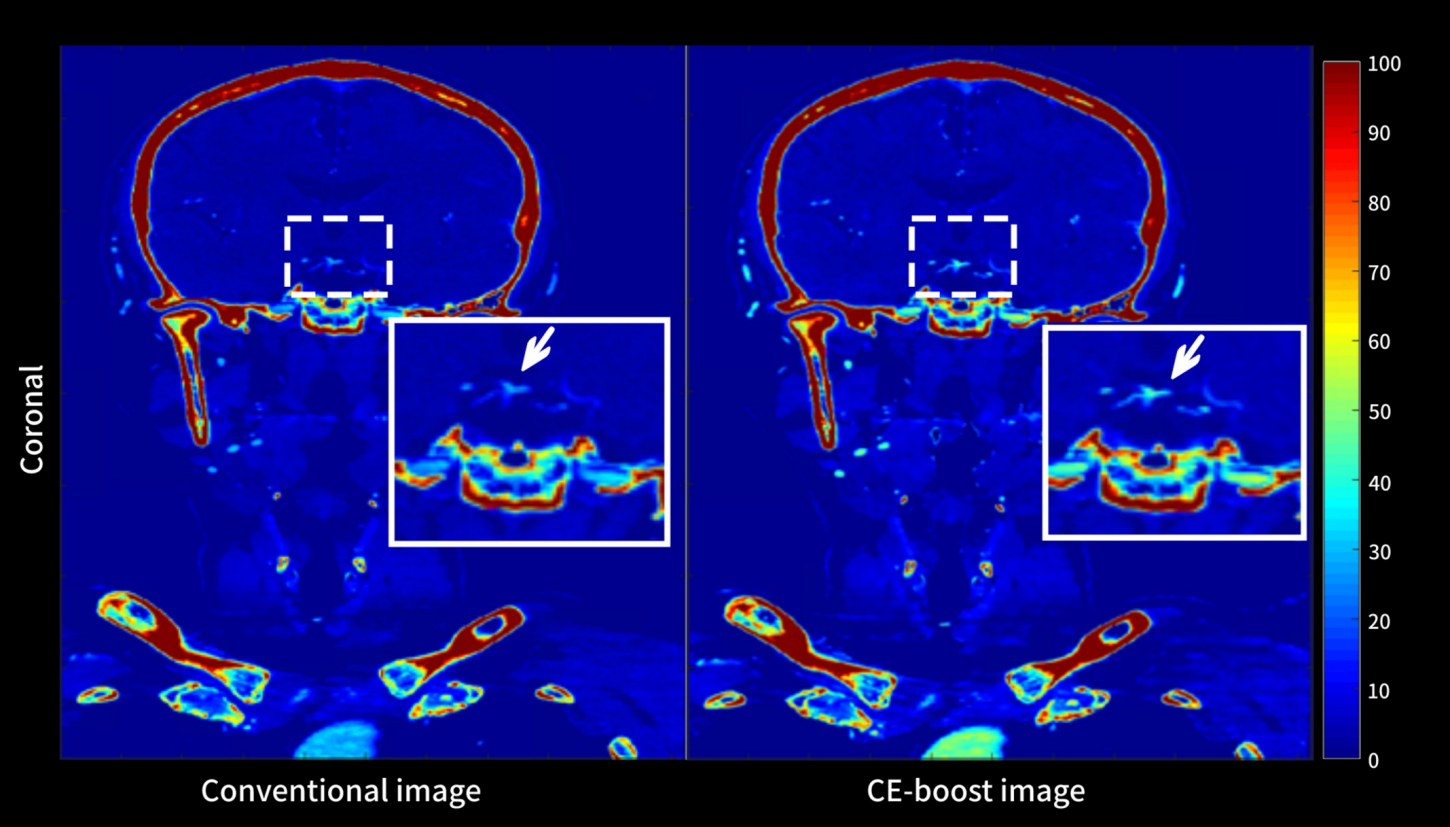
The SNR map between CE-boost and conventional images. CE-boost technique induced an improvement in the SNR values in the arteries of the posterior fossa especially in the basilar artery and superior cerebellar artery (white arrow) compared to the conventional image.

CE-boost technique yielded significantly (*p* < 0.001) higher CT attenuation than the conventional images from the different anatomical regions including PCA (649.13 ± 142.37 vs. 440.15 ± 91.68), BAs (665.63 ± 153.45 vs. 451.12 ± 94.05), pre-foraminal segments of the vertebral artery (665.64 ± 153.45 vs. 462.71 ± 103.51), foraminal segments of the vertebral artery (733.43 ± 161.88 vs. 508.66 ± 107.46), extradural segments of the vertebral artery (712.81 ± 165.36 vs. 494.62 ± 111.28), and intradural segments of the vertebral artery (674.04 ± 165.94 vs. 466.60 ± 112.12) (Fig 2). Fig 3 and Table 2 present the results of SNR and CNR. The mean value of SNR improved from 64.43 ± 17.17 to 121.37 ± 38.77, *p* < 0.001, when the CE-boost technique was applied on the conventional images. Moreover, the mean value of CNR was obviously higher for CE-boost images (116.65 ± 57.44) than for conventional images (56.90 ± 18.79) (*p* < 0.001). Fig 4 shows an illustration of the SNR map with CE-boost. The SNR map demonstrated that the higher SNR values in the arteries of the posterior fossa, especially in the BA and superior cerebellar artery, were obtained with CE-boost images than with the conventional image. Image noise was significantly (*p* < 0.001) lower for CE-boost images (6.09 ± 1.93) than for the conventional images (7.79 ± 1.73). The results of FMHW are shown in Table 3. The FWHM_pca_, FMHW_ba_, and FMHW_va_ was significantly shorter for CE-boost than for conventional images (*p* < 0.021, *p* < 0.001, *p* < 0.001). The FMHW_average_ was 2.81 ± 0.85 mm for CE-boost images and 2.91 ± 1.06 mm for conventional images (*p* < 0.001).

**Table 2:** The results of SNR and CNR: A comparison between CE-boost and conventional images.

	CE-boost images	Conventional images	*P* value
**Signal-to-noise ratio (SNR)**			
P1/P2–PCA	117.97 ± 57.02	54.76 ± 28.42	*0*.*001*
Basilar artery	112.40 ± 54.59	60.89 ± 42.79	*0*.*001*
V4–VA	123.24 ± 81.43	61.62 ± 35.04	*0*.*001*
V3–VA	137.57 ± 110.66	74.53 ± 38.54	*0*.*001*
V2–VA	117.55 ± 52.14	67.68 ± 30.71	*0*.*001*
V1–VA	119.51 ± 60.83	67.10 ± 27.93	*0*.*001*
**Average**	**121.37 ± 38.77**	**64.43 ± 17.17**	***0*.*001***
**Contrast-to-noise ratio (CNR)**			
P1/P2–PCA	108.49 ± 54.53	52.81 ± 17.87	*0*.*001*
Basilar artery	110.72 ± 53.95	54.10 ± 17.47	*0*.*001*
V4–VA	114.27 ± 56.23	55.84 ± 18.71	*0*.*001*
V3–VA	126.29 ± 61.21	61.90 ± 20.11	*0*.*001*
V2–VA	123.23 ± 62.60	60.17 ± 20.35	*0*.*001*
V1–VA	116.90 ± 61.40	56.54 ± 20.36	*0*.*001*
**Average**	**116.65 ± 57.44**	**56.90 ± 18.80**	***0*.*001***

Data are the mean ± standard deviation.

P1/P2–PCA, precommunicating/postcommunicating segments of the posterior cerebral artery; V4, pre-foraminal segment; VA, vertebral artery; V3, foraminal segment; V2, extradural segment; V1, intradural segments.

**Table 3:** The results of FMHW between conventional and CE-boost images.

	CE-boost images	Conventional	*P* value
PCA	2.16 ± 0.34 mm	2.18 ± 0.34 mm	*0*.*021*
BA	2.84 ± 0.46 mm	2.88 ± 0.48 mm	*0*.*001*
VA	3.60 ± 1.01 mm	3.87 ± 1.42 mm	*0*.*001*
**Average**	**2.81 ± 0.85 mm**	**2.91 ±1.06 mm**	***0*.*001***

Data are the mean ± standard deviation.

PCA, posterior cerebral artery; BA, basilar artery; VA, vertebral artery.

**Fig 5:**
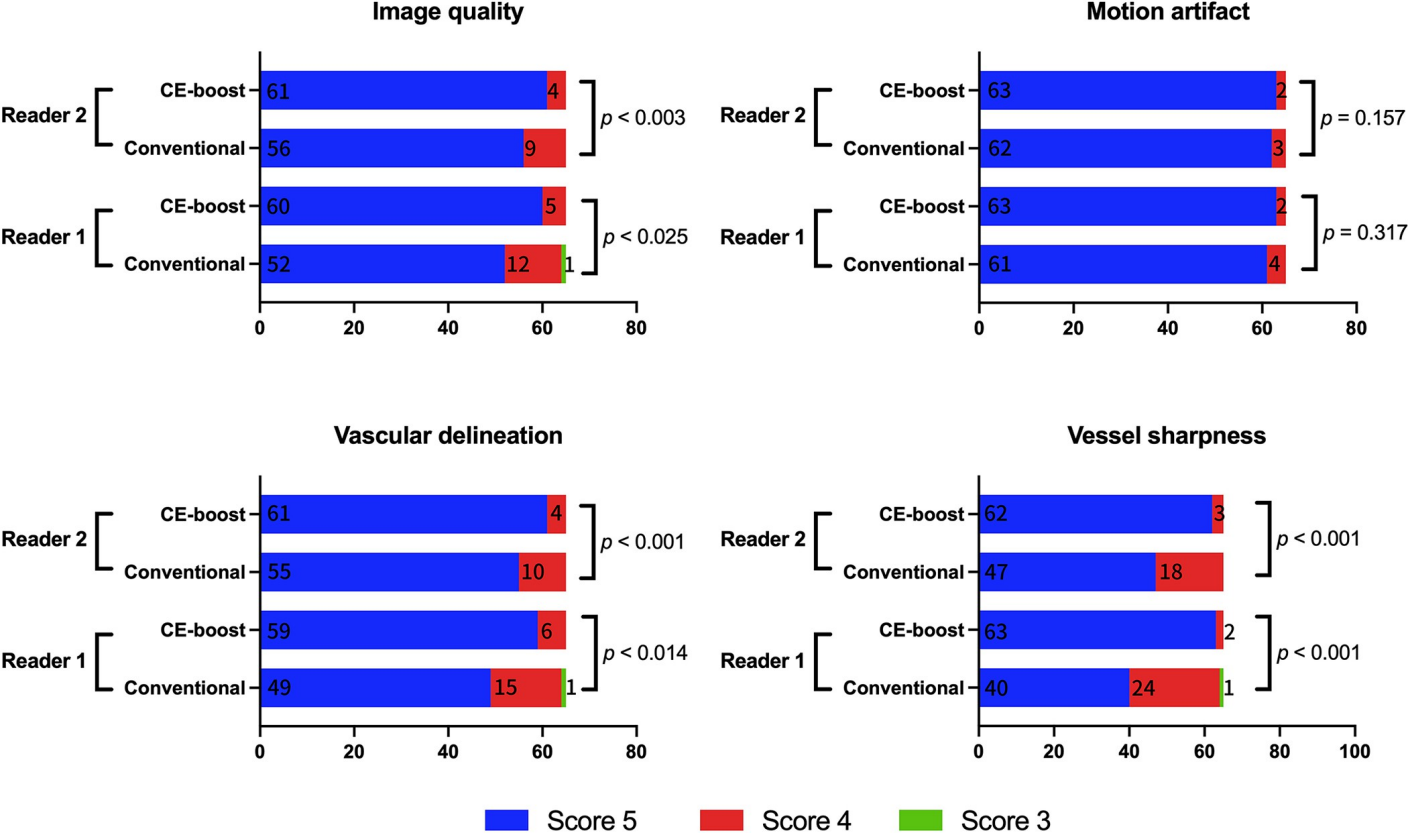
Results of the subjective image analysis between CE-boost and conventional images. CE-boost technique performed better than the conventional images in terms of overall image quality, vascular delineation, and vessel sharpness. There was no significant difference in the motion artifact between the conventional and CE-boost images. All CE-boost and conventional images were scored more than three scores by both observers.

**Fig 6:**
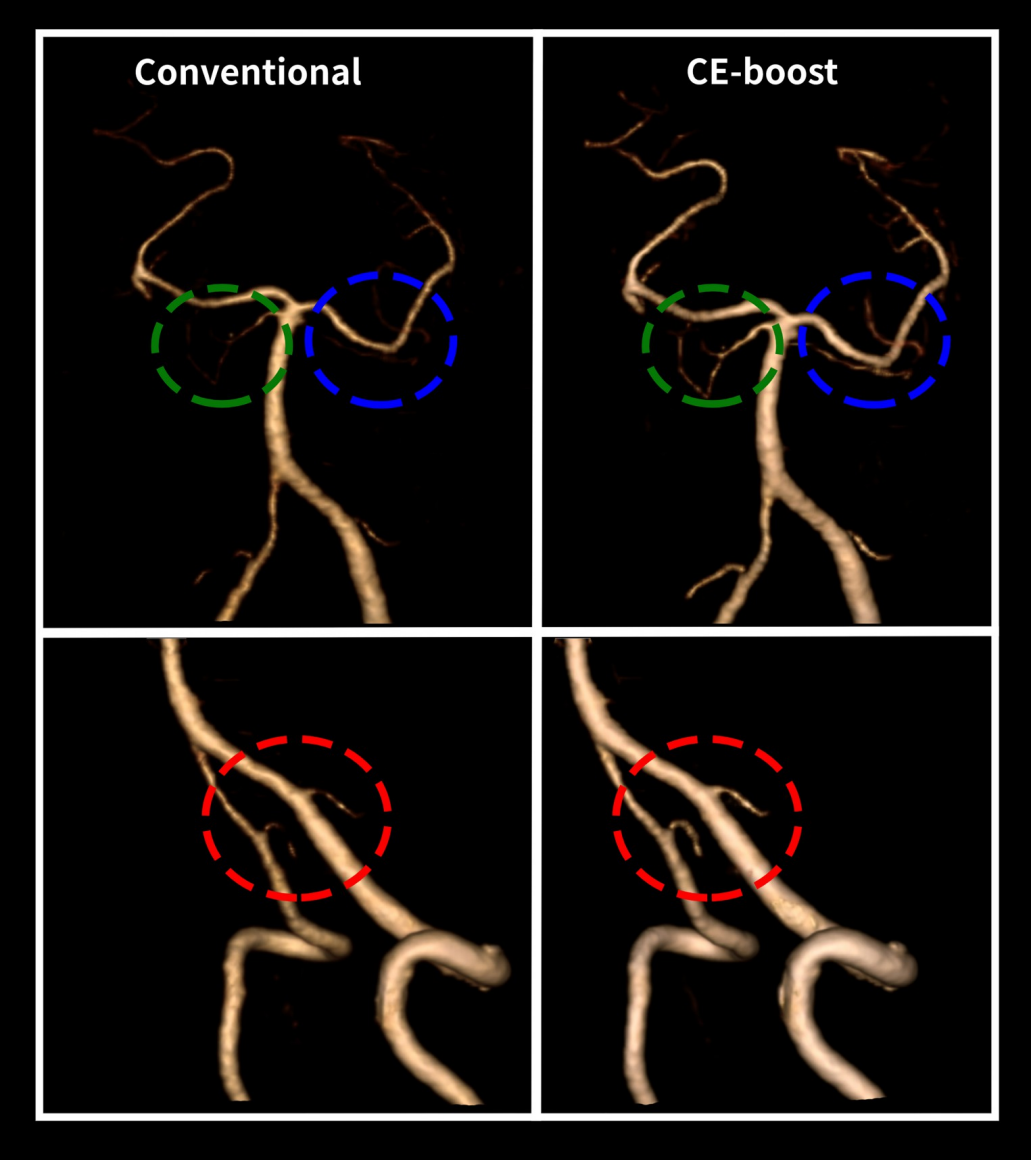
The volume-rendered images of the arteries in the posterior fossa. The CE-boost images showed clear visualization and improved completeness of the posterior inferior cerebellar arteries (red circle) and superior cerebellar arteries (green and blue circle) than conventional images on both sides.

**Fig 7:**
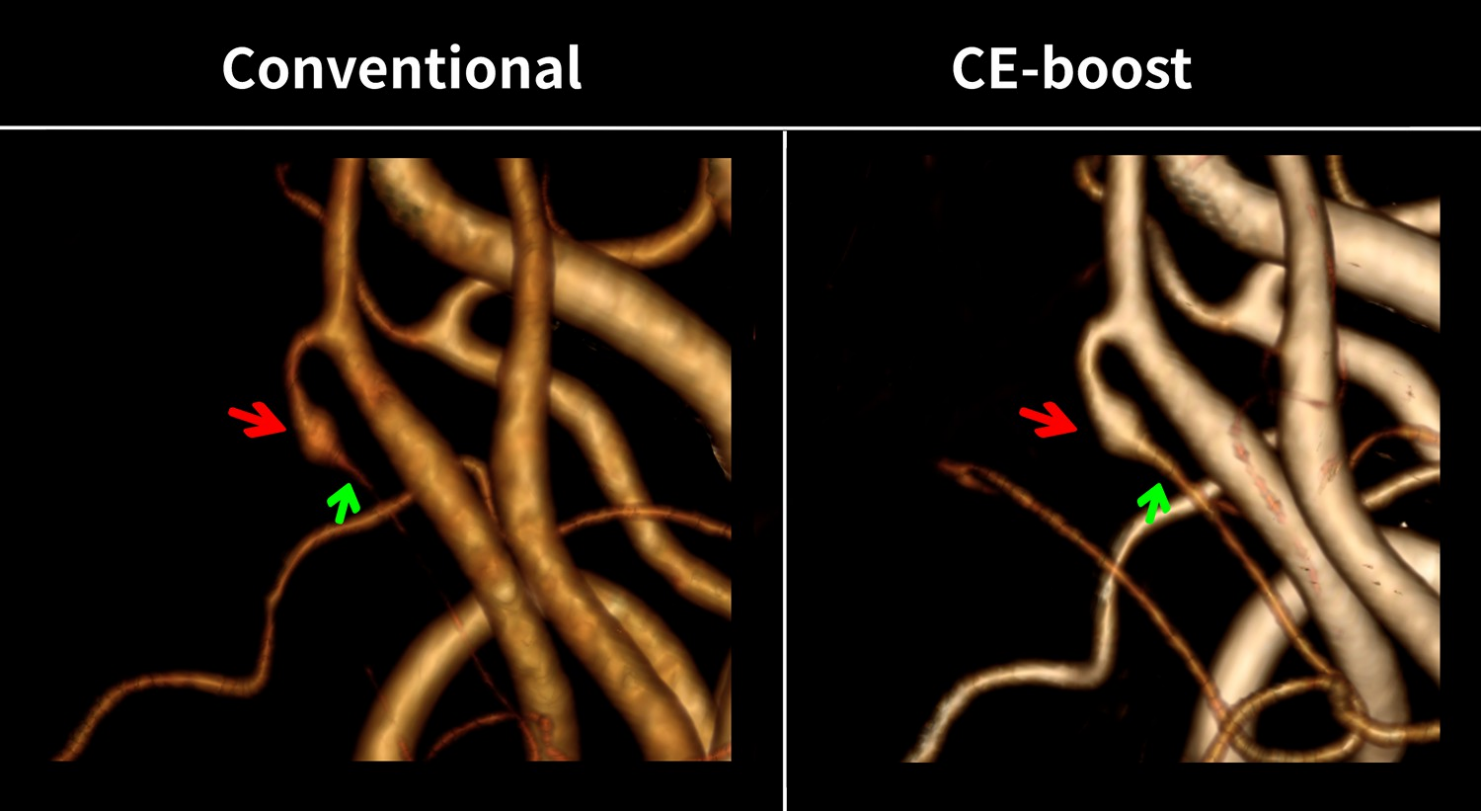
Representative case of a 60-year-old woman with a right posterior inferior cerebellar aneurysm. CE-boost showed a well demonstration of the distal segments (green arrow) of the right posterior inferior cerebellar artery distal to the aneurysm (red arrow) in a 3D angiogram compared to the conventional image. The values of the window width and level were the same between the two images.

The subjective image analysis scores obtained significantly higher scores with the CE-boost technique compared to conventional images with moderate inter-observer agreement (kappa: 0.58) (Fig 5). CE-boost images scored better than the conventional images in terms of overall image quality (4.93 ± 0.26 vs. 4.82 ± 0.40, *p* < 0.001) and vessel sharpness (4.96 ± 0.19 vs. 4.66 ± 0.49, *p* < 0.001). In addition, CE-boost performed higher vascular delineation (4.92 ± 0.27 vs. 4.79 ± 0.43, *p* < 0.001) than the conventional images. No additional motion artifacts were found in the CE-boost than in conventional images (4.97 ± 0.17 vs. 4.96 ± 0.23, *p* = 0.083). Moreover, vessel completeness was improved, and better arterial visualization in the posterior fossa was observed in the CE-boost images than in conventional images. The vessel delineation on the terminal branches of PCA was also significantly improved in the CE-boost images (Fig 6). Fig 7 shows the representative case of a posterior inferior cerebellar artery (PICA) aneurysm. Optimal demonstration of the distal segments of PICA was observed in CE-boost image than in conventional image.

## Discussion

In this study, head and neck CT angiography post-processing using CE-boost technique reveals a significant reduction in image noise and improvement in CT attenuation value, SNR, CNR, image sharpness, and subjective image quality compared with conventional images. To the best of our knowledge, this is the first study to investigate the CE-boost technique in head and neck CT angiography.

Iizuka et al. analyzed the CE-boost technique in an aortic CT angiography and concluded that the SNR and CNR were increased with CE-boost technique compared with conventional images as follows: 7.3 to 23.4 and 4.0 to 15, respectively [15]. Similarly, in this study, we found a higher SNR (121.37—CE-boost vs. 64.43—conventional image) CNR (116.65—CE-boost vs. 56.90—conventional image) in the head and neck CT angiography for CE-boost images than for conventional images. The result of our study also found that the depiction of intracranial small vessels has been improved with CE-boost technique. To prevent contrast-induced nephropathy, decreasing the flow rate and concentration of contrast media is recommended [20]. Unfortunately, decreasing the flow rate and concentration of contrast media results in a decline of vascular enhancement and decrease in the image quality in the CT angiography [21]. The effectiveness of the CE-boost technique in increasing the CT attenuation was observed compared with conventional images at the same concentration and flow rate of iodinated contrast media that could further decrease the concentration and flow rate of the iodinated contrast media while maintaining the image quality. Future studies are needed to investigate the image quality of CE-boost with the administration of lower amount of contrast media in the head and neck CT angiography.

The prevalence of anatomical variations is high in PICA and in the anterior inferior cerebellar artery, which are not often visualized using the conventional CT angiography owing to a low spatial resolution and the need for invasive DSA imaging for further analysis [22]. Although DSA is considered as the gold standard technique for the evaluation of vascular dissection, stenosis, aneurysm, and other vascular diseases with its high resolution, recently, it has been replaced by rapid and noninvasive CT angiography. Using CE-boost images on the head and neck CT angiography, this study resulted in better visualization of the PICA and superior cerebellar artery (SCA), which was not visualized in the conventional images. It is likely that the non-visualization of vessels could be explained by the insufficient degree of vascular opacity for the volume-rendering threshold by conventional images compared with CE-boost images [23]. In addition, FMWH is used to evaluate the and image sharpness [16]. In our study, FMWH was significantly shorter in CE-boost images than conventional images which reflects higher image sharpness with CE-boost. Conversely, regarding the higher vascular enhancement, the visualization of PICA and SCA vessels are improved in the CE-boost images. The vessel sharpness and vascular delineation were significantly higher in the CE-boost images than in the conventional images by both observers. Moreover, the identification of the variations and the evaluation of the origin of the vessels that was not visualized in the conventional images but was well demonstrated in the CE-boost technique will provide valuable information for endovascular treatment management and preoperative vascular mapping. In addition, the well demonstration of small vessels and distal segments of the vertebrobasilar arteries with CE-boost could curtail the unnecessary DSA procedure and prevent the procedural risks. In the literature, the movement-related motion artifact was reported with the use of CE-boost in the abdominal CT angiography [24]. In contrast, there is no involuntary motion in the head and neck CT angiography than in abdominal CT angiography. Both readers provided equivalent scores on the motion artifacts between the two methods. Thus, no additional motion artifacts were observed in the head and neck CT angiography with CE-boost images compared to conventional images.

The clear vessel delineation may sometimes be challenging owing to artifact and image noise in the posterior fossa and hinder the interpretation of the aneurysm or dissection in the vertebrobasilar system [25]. With the recent deep learning reconstruction, the image quality was significantly increased with a lower image noise and higher CNR, SNR, and spatial resolution, and the lesion detectability was also demonstrated to be better than the traditional filtered-back projection and iterative reconstruction methods [7,26,27]. The vertebrobasilar arteries were rated by both observers as having an excellent image quality in the CE-boost images than in conventional images. We also observed that the vessel completeness was markedly improved, and vessel delineation was clearer with CE-boost technique. This improvement in vessel boundaries and completeness indicates that the use of a CE-boost technique would allow optimal interpretation of various vessel pathologies.

This study has some limitations. First, this is a retrospective study and analysis was conducted in a small number of patients. Second, CE-boost technique requires both pre-contrast and contrast-enhanced images to obtain subtracted iodinated image. Some institutes may prefer not to scan the pre-contrast image in the head and neck CT angiography. However, in particular, pre-contrast images are often required to confirm the presence of intracranial hemorrhage [28]. Third, the diagnostic performance of CE-boost in the head and neck CT angiography was not performed in this study; only the objective and subjective image analysis was evaluated. We believe that a higher image quality and better comprehensive information on the vertebrobasilar arteries would lead to higher or competitive diagnostic performance in the CE-boost than conventional CT angiography. A further study is required to investigate the diagnostic performance of CE-boost. Despite these limitations, the CE-boost technique is the only post-processing technique that does not require additional hardware and is easy to use in routine clinical practice.

In conclusion, the CE-boost technique provided higher image quality in terms of both objective and subjective image analysis without increasing the flow rate and concentration of the contrast media in the head and neck CT angiography. CE-boost technique is more desirable for clinical use due to the improved completeness of the vessels and a much clearer visualization of the vessels in the posterior fossa compared to conventional images.
